# Association of orthostatic hypertension with mortality in the Systolic Hypertension in the Elderly Program

**DOI:** 10.1038/s41371-019-0180-4

**Published:** 2019-02-25

**Authors:** William J. Kostis, Davit Sargsyan, Choukri Mekkaoui, Abel E. Moreyra, Javier Cabrera, Nora M. Cosgrove, Jeanine E. Sedjro, John B. Kostis, William C. Cushman, John S. Pantazopoulos, Sara L. Pressel, Barry R. Davis

**Affiliations:** 1 Cardiovascular Institute, Rutgers Robert Wood Johnson Medical School, New Brunswick, NJ USA; 2 Athinoula A. Martinos Center for Biomedical Imaging, Department of Radiology, Massachusetts General Hospital, Harvard Medical School, Boston, MA USA; 3 Department of Statistics and Biostatistics, Rutgers University, Piscataway, NJ USA; 4 Memphis Veterans Affairs Medical Center, Memphis, TN USA; 5 University of Tennessee Health Science Center, Memphis, TN USA; 6 University of Texas Health Science Center, Houston, TX USA

**Keywords:** Risk factors, Hypertension

## Abstract

We examined the association of orthostatic hypertension with all-cause mortality in the active treatment and placebo randomized groups of the Systolic Hypertension in the Elderly Program (SHEP). SHEP was a multicenter, randomized, double-blind, placebo-controlled clinical trial of the effect of chlorthalidone-based antihypertensive treatment on the rate of occurrence of stroke among older persons with isolated systolic hypertension (ISH). Men and women aged 60 years and above with ISH defined by a systolic blood pressure (SBP) of 160 mm Hg or higher and diastolic blood pressure lower than 90 mm Hg were randomized to chlorthalidone-based stepped care therapy or matching placebo. Among 4736 SHEP participants, 4073 had a normal orthostatic response, 203 had orthostatic hypertension, and 438 had orthostatic hypotension. Compared with normal response, orthostatic hypertension was associated with higher all-cause mortality at 4.5 and 17 years in analyses adjusted for age, gender, treatment, SBP, and pulse pressure (PP, HR 1.87, 95% CI 1.30–2.69, *p* = 0.0007; HR 1.40, 95% CI 1.17–1.68, *p* = 0.0003, respectively). These associations remained significant after additional adjustment for risk factors and comorbidities (HR 1.43, 95% CI 0.99–0.08, *p* = 0.0566 at 4.5 years, and HR 1.27, 95% CI 1.06–1.53, *p* = 0.0096 at 17 years). The increased risk of all-cause mortality associated with orthostatic hypertension was observed in both the active and placebo groups without significant interaction between randomization group and the effect on mortality. Orthostatic hypertension is associated with future mortality risk, is easily detected, and can be used in refining cardiovascular risk assessment.

## Introduction

Upon standing, a significant proportion of blood volume pools in the legs and the splanchnic circulation of the lower abdomen. These changes are counterbalanced by activation of the autonomic nervous system including an increase in central sympathetic outflow [1–7]. Older individuals may have lower intravascular volumes, venous insufficiency, and impaired baroreflexes. As a result, orthostatic hypotension (oHypo) is not uncommon, and is associated with worse clinical outcomes including myocardial infarction, stroke, heart failure, cognitive impairment, and mortality [1–4, 6–8].

Orthostatic hypertension (oHyper), an increase in systolic blood pressure (SBP) upon standing, also occurs in older individuals. Townsend and associates reported that oHyper and oHypo occurred with similar rates (about 5%) among 8662 participants in SPRINT [9]. Orthostatic hypertension represents a clinically important condition that has also been observed in patients with diabetes, Parkinson’s Disease, renal vascular anomalies, and autonomic neuropathy [10–15]. Associations of oHyper with target organ damage include coronary heart disease, cerebrovascular disease, poorer scores on neurobehavioral function tests, more advanced white matter hyperintensities on CT or MRI, orthostatic intolerance, and chronic kidney disease [1, 14, 16–23]. Orthostatic hypertension is not usually appreciated by clinicians, and there are scant data on long-term clinical outcomes from placebo-controlled trials.

We examined the prevalence of oHyper, and its association with long-term all-cause mortality in the active treatment and placebo randomized groups at 4.5 and 17 years after randomization in the Systolic Hypertension in the Elderly Program (SHEP) [24–26].

## Methods

We performed post-hoc analyses using data from SHEP to investigate the relationship of oHyper to mortality. Details on the inclusion and exclusion criteria, sample size selection, blinding, randomization procedures, and statistical methods of the SHEP have previously been described [25]. We chose SHEP because of the availability of long-term information on mortality in the active and placebo groups (17 years). There are no other studies with long-term outcomes examining the effects of oHyper on all-cause mortality in patients randomized to active treatment or placebo. The placebo group allowed us to compare the effects of participants randomized to the active vs. the placebo group. Participants with isolated systolic hypertension (ISH, SBP ≥ 160 mm Hg, diastolic blood pressure [DBP] < 90 mm Hg) were randomized to chlorthalidone-based active treatment or matching placebo from March 1, 1985 to January 15, 1988 [24–26]. Mortality was ascertained from the National Death Index (NDI). Of the 4736 SHEP participants, 22 had incomplete records. Of the remaining 4714 participants, 2353 were randomized to active treatment and 2361 to placebo. SBP was measured twice by certified trained personnel after 5 min of quiet rest in the sitting position and after 1 and 3 min standing. The orthostatic change of SBP was defined as the difference between the average of the two seated measurements and the SBP after standing for 1 min at the baseline visit. Using the 3-min measurement yielded similar results. Orthostatic change from the sitting to standing position was categorized as hypertensive (oHyper, SBP increase by ≥15 mm Hg), hypotensive (oHypo, SBP decrease by >20 mm Hg), or normal (oNorm, changes in SBP between the other two categories). All participants gave informed consent to participate in SHEP.

### Statistical methods

All-cause mortality was ascertained at the end of the randomized phase of the clinical trial (4.5 years) and at 17 years following randomization (the maximum separation between the active treatment and placebo survival curves) [26]. Two Cox regression models on the relationship of oHyper to all-cause mortality were fitted. The first model was adjusted for age and gender since these variables are not affected by differences in lifestyle or disease, as well as for baseline SBP and pulse pressure (PP). The second analysis was adjusted for serum creatinine, diabetes, body mass index (BMI), smoking status, left ventricular failure, HDL cholesterol, and randomization to active treatment, as well as age, gender, and baseline seated SBP and PP. Analyses of the effects of oHyper compared to participants with normotensive response (oNorm), ascertained at baseline, on mortality at 4.5 years and at 17 years following randomization were performed using the intent-to-treat approach. These analyses were performed for the active treatment group, the placebo group, and for all participants combined.

The study was approved by the Institutional Review Boards of the University of Texas Health Science Center and the Robert Wood Johnson Medical School.

## Results

### Results in the active treatment and placebo groups of SHEP

The baseline characteristics of SHEP participants have been reported elsewhere [24, 25]. Out of 4276 participants included in these analyses, 1055 of 2140 participants (49.3%) in the active treatment group and 1090 of 2136 participants (51.0%) in the placebo group were dead at 17 years following randomization. The baseline characteristics of participants with oHyper compared to oNorm are reported in Table 1. Logistic regression identified the following variables as statistically significant predictors of oHyper: age (*p* = 0.005), BMI (*p* < 0.005), left ventricular failure (*p* < 0.005), and baseline SBP (*p* < 0.05).

**Table 1:** Baseline characteristics of SHEP patients with orthostatic hypertension as compared to those with normal orthostatic response

Predictor	oHyper	oNorm	Difference	*p* Value
*N*	203	4073	NA	NA
Active treatment	106 (52.2%)	2034 (49.9%)	1.16	0.3
Female	111 (54.7%)	2306 (56.6%)	0.95	0.776
Age	73.2 ± 7.2	72.1 ± 6.7	1.03	0.005
Creatinine	1.09 ± 0.3	1.06 ± 0.25	1.14	0.682
HDL	51.6 ± 12.3	53.1 ± 14.1	1.00	0.879
Diabetes	26 (12.8%)	407 (10%)	1.22	0.381
BMI	28.4 ± 4.9	27.1 ± 4.8	1.06	<0.0001
Smoking	29 (14.3%)	505 (12.4%)	1.39	0.129
LVF	25 (12.3%)	150 (3.7%)	3.38	<0.0001
Mean_Seated_SBP	168.3 ± 10.9	169.6 ± 11.6	0.98	0.024
Mean_Seated_PP	92.8 ± 16.5	92.7 ± 14.8	1.01	0.316
oSBP_1_min	−20.8 ± 7.3	4.0 ± 8.3	NA	NA
oPP_1_min	−10.4 ± 17.1	7.0 ± 11.3	NA	NA

### Factors associated with all-cause mortality in SHEP

**Fig. 1:**
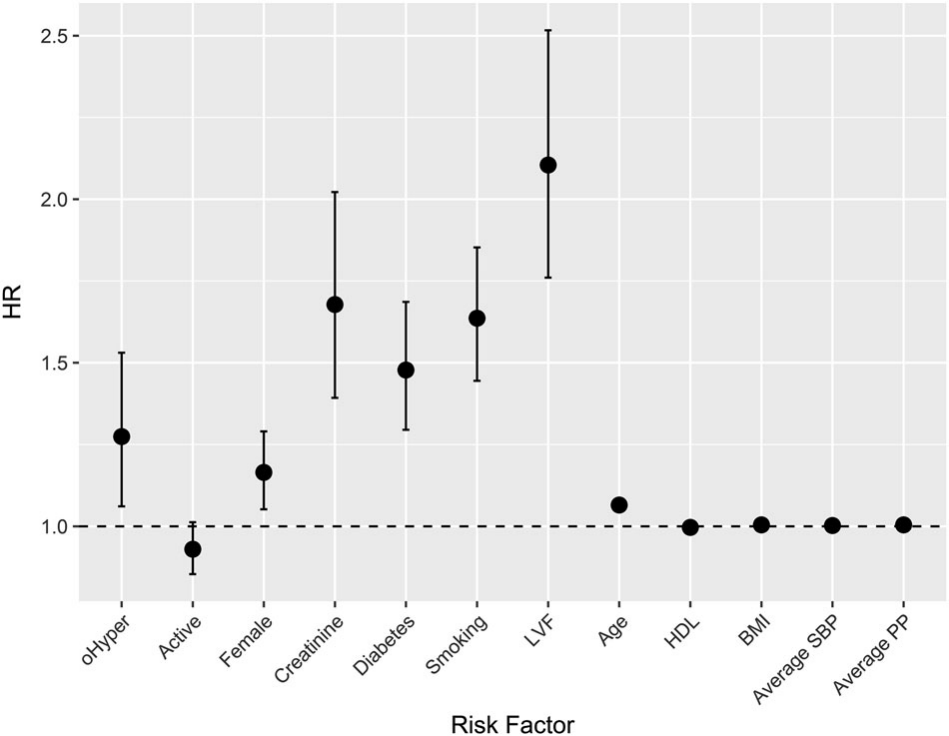
Factors associated with all-cause mortality at 17 years. Hazard ratios and 95% confidence intervals derived from a Cox regression model adjusting for demographic and clinical variables

Analyses adjusted for age, gender, creatinine, diabetes, BMI, smoking status, history of left ventricular failure, HDL cholesterol, average SBP at baseline, average PP at baseline, and randomization to active treatment, indicated a higher risk of death at 17 years for the following variables: female (HR 1.16, 95% CI 1.05–1.29, *p* = 0.0034), age (HR 1.06, 95% CI 1.06–1.07, *p* < 0.0001), creatinine (HR 1.68, 95% CI 1.39–2.02, *p* < 0.0001), diabetes (HR 1.48, 95% CI 1.30–1.69, *p* < 0.0001), smoking (HR 1.64, 95% CI 1.44–1.85, *p* < 0.0001), left ventricular failure (HR 2.10, 95% CI 1.76–2.52, *p* < 0.0001), and PP (HR 1.004, 95% CI 1.0004–1.0082, *p* = 0.0292). Randomization to the active treatment group was associated with lower risk of death (HR 0.85, 95% CI 0.76–0.96, *p* = 0.0098, Fig. 1).

### Effect of orthostatic change on all-cause mortality

After adjustment for age, gender, baseline SBP, and baseline PP, oHyper was associated with higher all-cause mortality at 4.5 years (HR 1.87, 95% CI 1.30–2.69, *p* = 0.0007) and at 17 years (HR 1.40, 95% CI 1.17–1.68, *p* = 0.0003). The association remained statistically significant after adjustment for serum creatinine, diabetes, BMI, smoking status, left ventricular failure, HDL cholesterol, and randomization to active treatment, as well as age, gender, baseline SBP, and baseline PP at 4.5 years (HR 1.43, 95% CI 0.99–2.08, *p* = 0.0566) and at 17 years (HR 1.27, 95% CI 1.06–1.53, *p* = 0.0096, Table 2).

**Table 2:** All-cause mortality at 4.5 and 17 years following randomization among patients with orthostatic hypertension as compared to normal orthostatic response

Follow-up interval (years)	Adjustment	*N*	Deaths	HR (95% CI)	*p* Value
4.5	None	4276	373	1.95 (1.36–2.80)	0.0003
4.5	Age, sex, SBP and PP	4276	373	1.87 (1.30–2.69)	0.0007
4.5	Multiple^a^	4276	373	1.43 (0.99–2.08)	0.0566
17	None	4276	2145	1.41 (1.17–1.69)	0.0002
17	Age, sex, SBP and PP	4276	2145	1.40 (1.17–1.68)	0.0003
17	Multiple^a^	4276	2145	1.27 (1.06–1.53)	0.0096

^a^Adjusted for age, gender, baseline seated SBP and PP, serum creatinine, diabetes, BMI, smoking, left ventricular failure, HDL cholesterol, and randomization to active treatment

### Effect of orthostatic change on all-cause mortality by randomized treatment

**Fig. 2:**
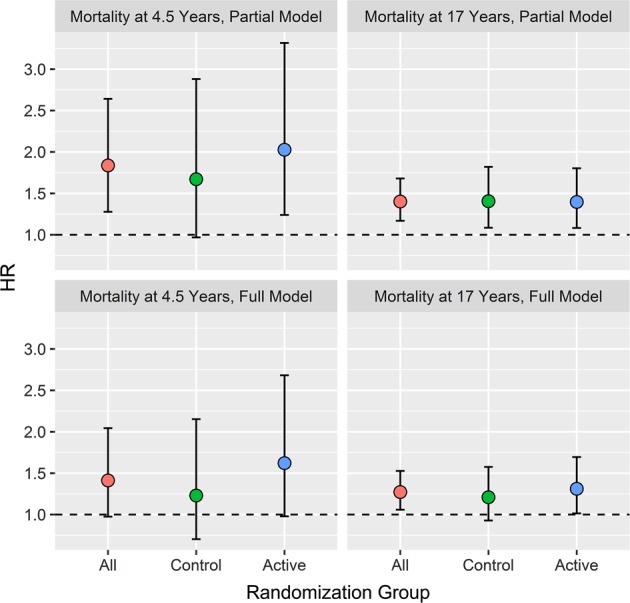
All-cause mortality at 4.5 and 17 years among patients with orthostatic hypertension as compared to normal response by randomization group (active treatment or placebo). Hazard ratios and 95% confidence intervals adjusted for age, sex, and systolic blood pressure, and pulse pressure (top) and adjusted for serum creatinine, diabetes, BMI, smoking status, left ventricular failure, HDL cholesterol, and randomization to active treatment, as well as age, gender, and baseline SBP and PP (bottom)

The increased risk of all-cause mortality associated with orthostatic hypertension was observed in both the active and placebo groups of SHEP (Fig. 2). When all participants were analyzed together, there was no significant interaction between randomization group (active vs. placebo) and the effect of oHyper on all-cause mortality (*p* for interaction = 0.5006 at 4.5 years and *p* for interaction = 0.8945 at 17 years) after full adjustment (adjustment for serum creatinine, diabetes, BMI, smoking status, left ventricular failure, HDL cholesterol, and randomization to active treatment, as well as age, gender, baseline SBP, and baseline PP).

## Discussion

The rate of orthostatic hypertension in SHEP (4.3%) is similar to that previously reported [9]. Participants with orthostatic hypertension were older, with higher SBP and BMI, and were more likely to have had a history of left ventricular failure. Orthostatic hypertension was associated with all-cause mortality after adjustment for age, gender, baseline SBP, and baseline PP. The associations remained statistically significant after additional adjustment for risk factors and comorbidities. Although all participants in SHEP were at least 60 years of age (mean 72 years), increasing age was associated with higher risk.

The mechanisms of orthostatic hypertension and the reasons it is associated with increased mortality are not fully understood. Orthostatic hypertension may be due to baroreceptor reflex abnormalities, increased vascular adrenergic activity, increased sensitivity of the cardiopulmonary and arterial baroreceptor reflexes, increased sympathetic activity, as well as stiffness of the central arteries [15, 27–29]. Studying pulse wave velocity and augmentation index, a measure of aortic stiffness, in 365 older hypertensives, Hoshide et al. observed that orthostatic hypertension was associated with altered aortic properties and with a higher augmentation index [22]. Also, patients with orthostatic hypertension exhibit an increase in PP upon standing unlike persons with normal response or oHypo [30].

The abnormalities associated with orthostatic hypertension listed above interacting with vascular aging (in addition to chronological age), risk factors such as smoking, and comorbidities such as renal disease and diabetes may cause increased risk of mortality. Autonomic dysregulation and abnormal baroreflexes that could be related to diabetes may also help explain some of the increased risk associated with orthostatic hypertension. Fedorowski and associates have identified excessive vascular adrenergic sensitivity, baroreceptor reflex abnormalities, and inappropriate activation of the renin–angiotensin–aldosterone system as potential mechanisms [15].

Exaggerated postural BP changes are associated with increased risk. In the Hypertension Detection and Follow-up Program (HDFP) and in the Honolulu Heart Program, orthostatic hypotension was associated with higher mortality in elderly men [1, 31]. Greater change of postural BP (either orthostatic hypertension or hypotension) was associated with increased risk of advanced silent brain lesions [32] and in the Framingham study, orthostatic hypertension was associated with arterial stiffness [33]. In a study by Kario et al., abnormal postural BP variation was associated with diurnal BP change in elderly hypertensive patients, where those with a marked nocturnal fall in blood pressure exhibited oHyper and those without it exhibited oHypo [34].

Additional studies have examined the association of orthostatic hypertension with mortality. In a community-based study, Velilla-Zancada and associates observed an association of systolic orthostatic hypertension with mortality although this association was not present after 1 min of standing, or at 1 or 3 min standing for DBP [35]. Veronese and associates reported an association of orthostatic hypertension with all-cause mortality among 2876 community-dwelling older participants at 4.4 years of follow up [36]. Taken together, the data from available studies are congruent with our findings in a much larger population with assessment of a longer-term outcome (mortality at 17 years after randomization). This study has limitations, however, including its retrospective nature, i.e., this analysis was not specified during the design of SHEP, the inclusion of only older patients with ISH who were otherwise healthy, using diuretic-based stepped-care therapy, and the lack of information on 24-h ambulatory blood pressure and measurement of orthostatic changes during the years after randomization. Also, there is no information on non-fatal events, pharmacologic therapy, and other interventions at 17 years following randomization. This study also has significant strengths, including the standardized BP measurements, the congruence of the effects at 4.5 years and 17 years, the 100% determination of vital status, and the very similar results in the active and placebo randomized groups. The latter point implies that oHyper is associated with increased mortality regardless of therapy. Thus, the findings presented here are relevant today although newer antihypertensive agents are now available in addition to those used in SHEP.

This study and previous reports associating orthostatic hypertension with increased mortality, indicate that orthostatic hypertension is an easily ascertained condition that can be used in predicting the risk of mortality. Although further research is needed in order to better understand the pathophysiology and epidemiology of orthostatic hypertension, it is reasonable to screen for this phenomenon as a risk factor in routine clinical practice as well as in clinical trials. Orthostatic hypertension is easily detected and can be used in refining cardiovascular risk assessment.

### Summary

#### What is known about the topic

- Orthostatic hypertension, an increase in SBP upon standing, occurs in about 5% of patients with hypertension.
- Orthostatic hypertension is an emerging risk factor for cardiovascular disease and is associated with hypertensive target-organ damage and cardiovascular events.
- Orthostatic hypertension is not usually appreciated by clinicians, and there are scant data on long-term mortality from placebo-controlled trials.

#### What this study adds

- In the Systolic Hypertension in the Elderly Program (SHEP), a double-blind placebo-controlled trial, participants with isolated systolic hypertension were randomized to chlorthalidone-based stepped care therapy or matching placebo and for which long-term mortality data are available.
- In analyses adjusted for age, gender, treatment, SBP, pulse pressure, risk factors, and comorbidities, orthostatic hypertension was associated with increased all-cause mortality at 4.5 and 17 years in both the active treatment and placebo groups.
- Orthostatic hypertension is easily detected and can be used in refining cardiovascular risk assessment.
